# Factors affecting mental fitness for work in a sample of mentally ill patients

**DOI:** 10.1186/1752-4458-3-25

**Published:** 2009-11-19

**Authors:** Yasser A Elsayed, Mohamed A Al-Zahrani, Mahmoud M Rashad

**Affiliations:** 1 Al-Amal Complex for Mental Health, Dammam, 31422, Saudi Arabia

## Abstract

**Background:**

Mental fitness for work is the ability of workers to perform their work without risks for themselves or others. Mental fitness was a neglected area of practice and research. Mental ill health at work seems to be rising as a cause of disablement. Psychiatrists who may have had no experience in relating mental health to working conditions are increasingly being asked to undertake these examinations. This research was done to explore the relationship of mental ill health and fitness to work and to recognize the differences between fit and unfit mentally ill patients.

**Methods:**

This study was cross sectional one. All cases referred to Al-Amal complex for assessment of mental fitness during a period of 12 months were included. Data collected included demographic and clinical characteristics, characteristics of the work environment and data about performance at work. All data was subjected to statistical analysis.

**Results:**

Total number of cases was 116, the mean age was 34.5 ± 1.4. Females were 35.3% of cases. The highly educated patients constitute 50.8% of cases. The decision of the committee was fit for regular work for 52.5%, unfit for 19.8% and modified work for 27.7%. The decision was appreciated only by 29.3% of cases. There were significant differences between fit, unfit and modified work groups. The fit group had higher level of education, less duration of illness, and better performance at work. Patients of the modified work group had more physical hazards in work environment and had more work shift and more frequent diagnosis of substance abuse. The unfit group had more duration of illness, more frequent hospitalizations, less productivity, and more diagnosis of schizophrenia.

**Conclusion:**

There are many factors affecting the mental fitness the most important are the characteristics of work environment and the most serious is the overall safety of patient to self and others. A lot of ethical and legal issues should be kept in mind during such assessment as patient's rights, society's rights, and the laws applied to unfit people.

## Background

Mental fitness to work is the ability of workers to perform their work without risks for themselves or others [1]. It is an important issue as same as physical fitness. Nevertheless, it is a neglected area of practice and research. Mental ill health at work seems to be rising as a cause of disablement [2]. Assessment of mental fitness is carried out to prevent future health and safety risks for the worker, coworkers and the public. With the increasing economic burden nowadays and increased awareness by rights of employees, assessment of mental fitness must be performed with great competence and objectivity; otherwise, the concerned subjects will feel unfairly treated and will distrust the outcome of the examination [3]. A good balance is needed between job opportunities, health and safety risks. The assessment of fitness for work is not a universal or static concept as there are a lot of factors that may impact it. Nevertheless, the absence of adequate training in this particular field and lack of any published guidelines, psychiatrists who may have had no experience in relating mental health to working conditions are increasingly being asked to undertake these examinations. Many psychiatrists sought empirical ways of assessment and decision making to achieve this mission and gained experience with time. Motivated by all the above issues, this study was done to; a) explore the relationship between mental illness and fitness to work, b) detect the important factors affecting mental fitness to work, c) identify the differences between fit and unfit mentally ill patients and lastly this study was a trial to translate the experience gained during years of work in such field into objective guidelines for assessment and decision making.

## Methods

This study was a cross-sectional one and was done in Al-Amal Complex for Mental Health, located in Dammam. The complex belongs to the Ministry of health, Kingdom of Saudi Arabia (KSA). The forensic psychiatry committee is one of the important units inside the complex. The committee has a dual obligation work, toward the patient and the referring agency. Among the reasons of referral to this committee are forensic problems, cases in need for legal guardian and assessment of mental fitness to work. Yearly, there are about 1000 cases received for assessment from different sources. The committee is operated by a multidisciplinary team. All the investigators are members in this team for many years. This study was approved by the scientific and ethical committee of Al-Amal Complex for Mental Health and informed consent was taken from the all subjects. All cases referred to the committee for assessment of mental fitness during a period of 12 months were recruited into the study. Data was obtained from interviews with patients themselves, caregivers, psychiatric files and reports from the employers. Information obtained included sociodemographic data, clinical variables, and history of treatment including previous hospitalization. Moreover, global assessment of function (GAF) scale [4] was applied for all subjects. To assess the work environment characteristics and behavior at work, the investigators included both subjective and objective data. The subjective data was obtained through application of some items from world health organization health and performance questionnaire (WHO-HPQ) [5] which was designed originally to provide information to employers about the indirect costs of untreated and under-treated employee's health problems. It is a brief self-report questionnaire that obtains information about sickness absence, productivity, critical incidents and work related accidents. The objective data was obtained through a list of items created by the investigators after reviewing the literature related to this topic [2,3,6,7]. The list included: level of productivity at work, level of disturbed relationships with colleagues, risk of physical hazards at work, level of public communications needed at work, degree of dealing with finances, degree of supervision over work, work load, and presence of disturbed behaviors at work. Each point was scored by the investigators low, intermediate or high according to information obtained from all sources listed above. Also, the list included; years of experience, distance between home and work in kilometers, presence of afternoon and night shifts, and absenteeism in the last year in days. All diagnoses were made according to mini-international neuropsychiatric interview (M.I.N.I.) which is a short structured diagnostic interview [8]. However, diagnoses of organic mental disorders and personality disorders were based on ICD-10 research diagnostic Criteria [9] and were validated by two consultant psychiatrists with good inter-rater reliability. In case of comorbidity, the investigators identified the primary diagnosis and comorbid diagnosis. Each subject was interviewed by the investigators at least once and some patients needed more sessions to finalize their assessment. At the first assessment visit, all patients were informed of 6 important issues; 1) who invited the committee to see him/her, 2) what the committee does professionally (team and different specialties), 3) the purpose of the examination. 4) What information the committee has already been given, 5) what is known and relevant to the report may not be regarded as confidential between patient and committee (the right of the committee to disclose information to employer), and 6) how the report may be used with or against the person. At the end of assessment, each subject was asked to rate his/her satisfaction level with decision of the committee and scored appreciating, not appreciating, or indifferent.

All the data was subjected to statistical analysis using SPSS version 10.0. The main findings are presented as proportions with 95% confidence intervals. According to the decision of the committee, the subjects were further classified into three groups; fit, unfit, and modified work groups. Comparisons were done using means, standard deviations, frequencies, Kruskal Wallis and ANOVA. Level of significance was detected at p value 0.05.

## Results

### Subject characteristics

Total number of cases recruited through 12 months duration was 234 cases, of which, only 116 cases agreed to give consent and their data was completed. The mean age was 34.5 ± 1.4 years. Characteristics of the sample and diagnoses are presented in table 1 and 2. The decision of unfit to work was temporary in 10 cases (43.5%) and permanent in 13 cases (56.5%)while the decision of fit for modified work was temporary in 21 cases (65.6%) and permanent in 11 cases (34.4%).

**Table 1:** characteristics of the subjects

Item		Results
**Age mean & SD**		34.5 ± 3.8

**Men n & %**		75 (64.7%)

**Females n & %**		41 (35.3%)

**Single n & %**		38 (32.8%)

**Married n & %**		78 (67.2%)

**Reason of referral**	**Disturbed behavior at work**	71 (61.3%)
	
	**Request from the patient**	45(38.7%)

**Education level**	**High**	59 (50.8%)
	
	**Intermediate**	32(27.5%)
	
	**Low**	25 (21.5%)

**Decision n & %**	**Fit**	61 (52.5%)
	
	**Unfit**	23 (19.8%)
	
	**Modified work**	32 (27.7%)

**Satisfaction with the decision**	**Appreciated**	34 (29.3%)
	
	**Not appreciated**	66 (56.9%)

n, number of subjects; %, percentage; SD, standard deviation

**Table 2:** Current diagnoses

Diagnosis	n & (%)
**Schizophrenia and delusional disorders**	17 (14.7%)

**All Subsances of abuse**	24 (20.7%)

**Amphetamine**	18 (15.5%)

**Major Depressive disorder**	16 (13.8%)

**Bipolar disorder**	5 (4.3%)

**Anxiety disorders**	15 (12.9%)

**Social phobia**	12 (10%)

**Adjustment**	11 (9.5%)

**Conversion and dissociation**	3 (2.6%)

**Somatoform disorders**	4 (3.4%)

**Personality disorders**	10 (8.6%)

**Organic mental disorders**	3 (2.6%)

**Malinger**	8 (6.9%)

**Comorbidity mental and physical**	49 (42.2%)

n, number of subjects; %, percentage

Comorbidity was found in 49 cases; psychiatric comorbidity in 34 cases (29.3%) and physical comorbidity in 15 cases (12.9%).

There were significant differences between fit, unfit, and modified work groups in a number of parameters as shown in table 3, 4 and 5. There were no statistically significant differences between groups as regard GAF, working hours, work load, deal with finances, years of experience and number of workers supervised by the patient.

**Table 3:** comparisons between groups

Characteristics	Fit n 61	Unfit n 23	Modified n 32	P value
**Age in years, mean ± SD**	31.88 (11.6)	39.58 (10.5)	30.19 (9.4)	0.043

**Education, mean ± SD**	12.9 ± 2.61	7.5 ± 3.51	9.4 ± 2.8	0.05

**Duration of illness years, mean ± SD**	3.8 ± 1.92	10.12 ± 2.35	4.2 ± 3.41	0.01

**Hospital days, mean ± SD**	9.03 ± 4.15	58.9 ± 18.21	15.03 ± 12.04	0.001

**Age at onset, mean ± SD**	23.0 (8.9)	19.0 (8.6)	23.5 ± 6.5	0.062

**Hospitalized in past year, % & (n)**	26.2% (16)	56.5% (13)	31.2% (10)	0.05

**Absenteeism at last year in days, mean ± SD**	24.6 ± 3.13	66.4 ± 2.13	33.2 ± 3.41	0.01

**High Physical hazards at work, % & n**	18% (11)	47.8% (11)	68.8%(22)	0.03

**Presence of afternoon and night shifts, % & n**	31.1%(19)	43.5%(10)	56.2% (18)	0.05

**The work need high level of communication with public, % & n**	19.8% (12)	34.8% (8)	15.6%(5)	0.05

**High level of Disturbed Relationship with colleagues, % & n**	47.5%(29)	78.3%(18)	50% (16)	0.05

**Low Productivity at work, % & n**	32.9% (20)	91.3%(21)	59.4% (19)	0.05

**Years of experience, mean and SD**	14.4 ± 4.5	17.3 ± 3.6	13.5 ± 2.9	0.554

n, number of subjects; %, percentage; SD, standard deviation

P value is significant at ≤ 0.05

**Table 4:** Items from WHO health and work performance questionnaire

Characteristics	Fit n 61 (%)	Unfit n 23 (%)	Modified n 32 (%)	P value
**An accident or event that caused either damage, work delay, a near miss, or a safety risk in the last month**	18(29.5%)	12 (52.2%)	20(62.5%)	0.04

**An experience of work failure at the last month**	38 (62.3%)	8(34.8%)	22(68.8%)	0.05

**doing no work at most of the work time**	13(21.3%)	18 (78.3%)	17(53.1%)	0.03

**Work performance at last year ≥ 7 out of 10**	7 (11.5%)	15 (65.2%)	5 (15.6%)	0.003

**Better overall job performance during the past year in comparisons to other workers**	5 (8.2%)	16 (69.6%)	4 (12.5%)	0.001

**Worse overall job performance during the past year in comparison to other workers**	50 (82%)	3 (5%)	23 (71.9%)	0.001

n, number of subjects; %, percentage

P value is significant at ≤ 0.05

**Table 5:** comparisons between groups as regard diagnoses and drugs received

Characteristics	Fit n 61	Unfit n 23	Modified n 32	P value
**Substance % & (n 24)**	29.1% (7)	16.6% (4)	54.2% (13)	0.004

**Schizophrenia % &(n 17)**	29.4% (5)	47% (8)	23.6% (4)	0.005

**Depressive disorder % (n 16)**	62.5% (10)	12.5%(2)	25% (4)	0.001

**Adjustment % (n 11)**	90.9% (10)	00	9.1% (1)	0.001

**Personality disorders % (n 10)**	80% (8)	10% (1)	10% (1)	0.001

**Comorbidity % (n 49)**	20.4% (10)	46.9% (23)	32.7% (16)	0.02

**Antidepressants drugs % & (n)**	65.6%(40)	17.4%(4)	56.%(18)	0.01

**Typical antipsychotic drugs % & (n)**	4.9%(3)	43.5%(10)	6.3%(2)	0.005

**Use of Mood stabilizers % & (n)**	16.4% (10)	17.4% (4)	15.6% (5)	0.241

**Use of atypical antipsychotic drugs % & (n)**	29.5% (18)	34.8% (8)	31.3% (10)	0.064

**Use of polypharmacy % & (n)**	67.2% (41)	73.9% (17)	59.4% (19)	0.445

n, number of subjects; %, percentage

P value is significant at ≤ 0.05

All patients diagnosed as conversion or somatoform disorders and all malingerers were fit. There were no significant differences between groups regarding the following diagnoses; anxiety disorders, bipolar disorder, and organic mental disorders. Also, there were no statistically significant differences between groups as regard duration of treatment, use of mood stabilizers, atypical antipsychotic drugs or polypharmacy.

## Discussion

Assessment of mental fitness for work is considered in three main conditions; return to work after being off work for a period of time due to mental illness, request from the employee or employer for assessment and lastly recruitment of new staff. The current study has focused on the first and second conditions because they are the most sensitive and the most commonly referred cases to the forensic committee. In this study, the goal of assessment of each subject was to reach one of three decisions either fit for regular work, unfit for work, or lastly fit for wok with certain modifications in the work environment. The forensic committee took the decision of fitness to work according to consensus of all members of the multidisciplinary team including the investigators that's why this study is a trial to translate this experience into guidelines that are as objective as possible for such assessment and decision making.

The ways in which subjects are assessed for mental fitness are always questionable as there are conflicts of interest in the sources of data (the patient and the employer). That's why the investigators listened to patients and asked the employers for reports and put their own assessment of the work environment characteristics. Nevertheless, 56.9% of the subjects didn't appreciate the decision of the committee. This is an important aspect of dual obligation work as always there is unsatisfied side. Lack of appreciation may lead in many cases to lawsuits against the team.

### Characteristics of the sample

The mean age of the subjects referred was 34 ± 3.8 years. This age is the most productive time of the person's life [10]. It means that each subject has at least an average 25 years till the age of retirement which makes the decision of unfit to work difficult and expensive decision. Men constitute 64.7% of the sample and women 35.3%. This difference is not significant because the rate of employment for women is much less than employment for men in KSA [11]. Also, 32% of the cases were single which may be attributed to delayed marital age especially in mentally ill patients. Although the sample included subjects from all educational levels, 50.8% of referred cases were highly educated. This sounds logic as subjects who are highly educated usually work in important positions and are more easily detected when they become mentally ill [12]. While mentally ill patients with low educational level usually take longer time to be detected because they commonly work in simple jobs.

Substance abuse and dependence was the most prevalent diagnosis (20.7%) among the referred sample. These patients are usually disturbing for others, irresponsible and have a lot of troubles at work [13-15]. Moslem's culture strictly prohibits use of all substances including social drinking. That's why many employers can tolerate presence of some mentally disabled workers but never to accept substance users.

The second common diagnoses were schizophrenia and delusional disorders (14.7%). This may be explained by failure of schizophrenic subjects to adapt to their work. Their disturbed behaviors are easily detected and consequently they lose their jobs. Schizophrenics are among the most discriminated against of all disabled people. In the current study, unfit schizophrenic cases constituted 47% which is similar to the rate of unemployment among schizophrenics in a British study [15].

The most commonly presented anxiety disorder was social phobia (n 12 = 10% of the sample). This rate is accepted due to increased rate of social phobia reported in Saudi Arabia [16]. The rate of major depressive disorder was unexpectedly low (13.8%) in comparison to western studies of functional impairment of mentally ill people [13,17]. This result may be explained culturally as many cases of depression respond well to traditional treatments and adapt easier than patients with schizophrenia, substance abuse or social phobia.

There was a high rate of comorbidity (42.2% of cases) which was consistent with Gerkin's study [18] who reported that combinations of mental ill health, substance misuse, and chronic physical illness produce more disability days than would be predicted by adding their component effects.

Despite the majority of mental patients in this sample were addicts and schizophrenics, yet, the tendency of committee decision was towards fitness to work (either full fitness or modified fitness) more than unfitness. This may emphasize that having mental illness in a subject doesn't necessarily indicate that he/she is unfit to work. It further points to the notion that there are other capability factors that affect the ability of the person to work rather than just having mental illness per se.

### Comparisons between groups

The productivity of all groups was impaired but relatively the fit group had less impairment than other groups. The most common psychiatric disorders reported among the fit group are adjustment, personality and depressive disorders with the least comorbidity rates. While the unfit group consisted mainly of schizophrenic subjects and reported the highest comorbidity rates. Meanwhile, the modified fitness group was mainly substance use subjects. These findings suggest that the type of psychiatric diagnosis may have direct relation to fitness to work. Furthermore, Bullying is often identified [15] especially in malingerers and minor psychiatric disorders. This explains why 100% of subjects with conversion and somatoform disorders in addition to malingerers in the current study were given fit decision.

**The fit group **had significantly shorter duration of illness, younger mean age, shorter mean hospitalization time, less frequent hospitalization in the last year and less frequent absenteeism. These findings could be simply explained by differences in the stage of illness that might disappear after few years of chronicity. Also, these findings might indicate less severity of illnesses, hence less impairment of function. Moreover, the fit group was significantly more educated, had little physical hazards in their works, and little afternoon or night shifts. These data appear logic as educated people have more reserve and more power of adaptation to illnesses [19] and usually work in better positions with less physical hazards and work is only one morning shift.

Interestingly, 40 cases of **the fit group **used antidepressant drugs while actually only 25 cases diagnosed with depression and anxiety disorders. This is explained by either increased use of antidepressant drugs in cases like conversion, substance abuse and adjustment disorders or due to actual improvement in function with use of antidepressants [20].

**The unfit group **had more duration of illness, more frequent hospitalizations in the last year, more hospitalization days, more need for communication with the public during work, more disturbed relationship with their colleagues, less productivity, more comorbidity and more diagnosis of schizophrenia. These results seem to be related to each other. In contrast to the fit group, **the unfit group **had less use of antidepressant drugs and more use of conventional antipsychotic drugs which may be due to either overrepresentation of subjects with psychosis in the group or actual impairment in function by conventional antipsychotic drugs and lack of antidepressant drugs [21].

The decision of **unfitness to work **was either temporary or permanent. Temporary unfit means the patient will be given sick leave and reassessed again after suitable time. Permanent unfit is the most difficult decision as it means that the employee will never be fit for the job and that employee is unable to do any available job, with or without work modifications.

On the other hand, the decision of **work environment modification **was given to those patients who had controversies between the two other groups. This group significantly had more physical hazards in work environment, more afternoon and night shifts and more frequent diagnosis of substance abuse and dependence. Actually, diagnosis of substance abuse is a serious diagnosis hindering any work as patients may abuse drugs during work and became dangerous to themselves and others. It is worth mentioning that the commonest substance of abuse in the current study and in the region was amphetamine, with relatively high rate of amphetamine induced psychosis [22]. Also, a judgment in this category means the employee would be a hazard to self or others if employed in the job as described by his/her employer but would be considered fit to do the job if certain working conditions were modified. The modifications required must be clearly described. Again, the decision is either temporary or permanent. Temporarily means that if the person's condition improves with time, the decision may be lifted. Permanent work modification means that the employee will never be fit for the job without these modifications. Unfortunately, there is no uniformly applicable psychiatric wheelchair ramp [3]. **Modifications might include **shortening of working hours, reduction of work load, graded resumption of responsibilities, working away from physical hazards or providing strict supervision for those who abuse substances [23,24]. Nevertheless, work modification may interfere with the schedule of others and add to their workload and may lead to further discrimination by co-workers. That's why this decision should be time limited as much as possible.

Surprisingly, it was found that 65.2% from the unfit group scored 7 or more out of 10 for their job performance during the last year in comparison to 11.5% and 15.6% for the fit group and the modified work group respectively. Also, 62.3% of the fit group and 68.8% of the modified work group had an experience of work failure in the last month while only 34.8% of the unfit group had the same experience. These results express the way each subject evaluates him/herself and the degree of the discrepancy between the decision of the committee and patients' satisfaction and expectations. They also reflect to what extent subjects may distrust such assessment. For instance, most unfit schizophrenics have high expectations to work, in contrast to most of fit non psychotic patients. The same issue was repeated again in answer to a question "how would you compare your overall job performance during the past year with the performance of other workers who have a similar type of job? The answer was better than other workers in 69.6% for the unfit group. This indicates that self rating questionnaires are not valid or reliable ways to assess mental fitness. Another finding from the WHO health and work performance questionnaire was that 52.2% of the unfit group and 62.5% of the modified work group had an event that caused either damage, work delay or safety risk in the last month. This finding supports the decision of the committee. Moreover, in answer to the question "How often did you do no work at times when you were supposed to be working?" 78.3% of the unfit group found themselves doing no work when they were supposed to be working. This denotes the degree of tolerance in the work environment, either these patients don't really work or their supervisors don't rely upon their work most of time, which again supports the decision of the committee.

**There are many other factors **that are difficult to measure and should be considered in assessing mental fitness to work. One important factor is the ethical factor, balancing between patient's rights, employer's rights and society's rights. Another factor is the impact of decision on patients' lives like the maximum duration of sick leave that can be given, the extent to which patients will be compensated if they become unfit for work and the social influences on the employee upon given this decision. Lastly, there are a lot of cultural differences in tolerating mentally ill people in different jobs. Generally, the Arab culture is more tolerant for mentally ill people and the concept of work productivity is not clear for workers or employers especially in governmental jobs [25] which again explain why 78.3% of the unfit group found themselves without work most of the time in the last year. In addition, Arab countries usually allow for over-employment [26] in simple jobs, hence productivity is compromised even in healthy people. That's why the fitness decision is related to the culture, the place of work and the nature of employer.

Although there seems to be a growing interest in the field of assessment of fitness for work [27], yet, the literature is still very scarce and rarely based on experimental design [28]. One of the difficulties encountered in this study is the unclarity of research designs in this area. This could probably be owing to the complexities of such assessment with regard to its conceptual constraints, ethical implications and difficulties related to methodological aspects. Many subjects refused to give consent for the study which represented a challenge for such kind of research. One of the problems that faced the investigators is the underdevelopment of risk management strategies in different associations in addition to lack of occupational health staff which made the communication with employers difficult and time consuming. Also, data presented to the committee team usually deficient and incomplete. Moreover, lack of satisfaction of patients is an important problem in this field. Another limitation is that the current study didn't pay attention to the level of stress outside the work which is an important influential factor. Although this study presents a general cross-sectional design, the tools used are used for the longitudinal evaluation of past functioning. It would be interesting to take into account the fact that retrospective data might be memory biased. Occupations were not listed in the results as the investigators gave more importance to characteristics of work environment and found this easier to do comparisons. Many occupations were presented in the current study as teachers, soldiers, engineers, technicians, machinists, nurses, etc. But the number of subjects in each occupation was too little to do analytical statistics. However, there are many influential factors related to occupation and need attention in separate studies.

## Conclusion

There is a general belief that the psychiatrist should find the balance between loyalty to the patient and the duty of employers to offer safe effective service. System with greater communication, cooperation and understanding between the psychiatrist, the personnel department, the supervisor and other health professionals is strongly recommended but confidentiality is often reported as a barrier. Legislation shows that employers need to be aware of the relapsing and remitting nature of mental disorders and the potential for adjustments. Psychiatrists should bear in mind that the validity and effectiveness of judgments on unfitness for work are still doubtful and inconsistent. Standardized criteria are strongly needed and enabling options should always be considered.

The assessments of fitness for work should focus on job requirements more than focusing solely on medical diagnoses. So before assessment of the patient, data about characteristics of working environment must be available. The most specific and important information according to the current study were level of physical hazards, presence of afternoon and night shifts, level of public communication needed, relationship with colleagues, productivity and absenteeism. However, other factors cannot be excluded by only one study on 116 cases. That's why factors like working hours, work load, leadership skills needed, work in finances, duration of service, job location and degree of supervision should be considered. At the same time, the criteria used to evaluate fitness should include ethical, economic and legal considerations. Being unemployed is associated with high level of psychiatric morbidity as the working environment can be an important determinant of both mental ill health and, for many, wellbeing.

## Competing interests

The authors declare that they have no competing interests.

## Authors' contributions

All authors conceived of the study and participated in its design and coordination. YAE administered the instrument and collected the data. MAA directed and oversaw the statistical analysis. MMR participated in data collection and conducted statistical analysis. All authors participated in the writing and revision and approved the final manuscript.
